# Generalization in Adaptation to Stable and Unstable Dynamics

**DOI:** 10.1371/journal.pone.0045075

**Published:** 2012-10-08

**Authors:** Abdelhamid Kadiallah, David W. Franklin, Etienne Burdet

**Affiliations:** 1 Department of Bioengineering, Imperial College of Science, Technology and Medicine, London, United Kingdom; 2 Computational and Biological Learning Laboratory, Department of Engineering, University of Cambridge, Cambridge, United Kingdom; The University of Western Ontario, Canada

## Abstract

Humans skillfully manipulate objects and tools despite the inherent instability. In order to succeed at these tasks, the sensorimotor control system must build an internal representation of both the force and mechanical impedance. As it is not practical to either learn or store motor commands for every possible future action, the sensorimotor control system generalizes a control strategy for a range of movements based on learning performed over a set of movements. Here, we introduce a computational model for this learning and generalization, which specifies how to learn feedforward muscle activity in a function of the state space. Specifically, by incorporating co-activation as a function of error into the feedback command, we are able to derive an algorithm from a gradient descent minimization of motion error and effort, subject to maintaining a stability margin. This algorithm can be used to learn to coordinate any of a variety of motor primitives such as force fields, muscle synergies, physical models or artificial neural networks. This model for human learning and generalization is able to adapt to both stable and unstable dynamics, and provides a controller for generating efficient adaptive motor behavior in robots. Simulation results exhibit predictions consistent with all experiments on learning of novel dynamics requiring adaptation of force and impedance, and enable us to re-examine some of the previous interpretations of experiments on generalization.

## Introduction

When learning a novel motor task, it is not possible to either practice, or store motor commands, for every possible future motor action. This means that the CNS must learn to generalize control strategies from the small set of trained movements at its disposal. Studies which have investigated generalization in learning a stable interaction with a novel dynamic environment have demonstrated the formation of an internal representation of the motor commands necessary for feedforward control [1]–[5]. The learning of this internal representation on a single movement affects the control strategy in neighboring regions [1], [6]–[8]. These studies in generalization have demonstrated that motor learning is not rote memorization, but due to an internal representation which depends on, and generalizes over, the state space [2], [9]. However, the broad size of the estimated basis functions, through which generalization is proposed to occur, could prevent the learning of force fields with fine granularity. Therefore, the successful learning of such fine grained force fields was interpreted as evidence that the breadth of neural basis functions were adapted to the complexity of the experienced environment [3].

An early model capable of both increasing stiffness and force was developed by Loeb [10], [11]. This model, consisting of a single joint with realistic antagonist muscles, sensor dynamics and spinal cord pathways, was tested on several tasks including external perturbations. As this model's parameters were obtained in one batch through numerical optimization, the model did not explicitly represent mechanisms for motor learning. Models of motor learning [7], [8], [12]–[16], some of which generalize in state space, are able to predict the evolution of force for learning stable dynamics. However, these models do not consider the effects of noise due to motor output variability [17] and the interaction of this variability with the environment, nor do they possess a mechanism to deal with unstable situations typical of tool use [18], [19]. Furthermore, the evolution patterns of muscle activation in novel stable dynamics predicted by these models are different from the patterns observed in experiments [20] in which co-activation is found.

Recently we have proposed a model of motor learning in muscle space which adapts and coordinates the temporal muscle activations patterns such that it simultaneously minimizes instability and the effects of noise, adapts to changes in the dynamics of the body and the environment, and minimizes the metabolic cost [21]. This model was demonstrated to adapt a two-joint six-muscle biomechanical model of planar arm motion to stable and unstable dynamic interactions [21], [22], but the algorithm had no capability for generalization and was demonstrated only along a single movement, as a function of time rather than over a state space. Simulations were performed along only a single movement, which was performed simply using a one degree-of-freedom look-up-table as a function of movement time to learn the feedforward motor command. This feedforward motor command was updated after each trial using a V-shaped function of the kinematic error, such that motor command activity increased when the muscle was either stretched or shortened, but decreased when the muscle was close to the desired length. This function specifies, based on the movement error of the previous trial, the manner in which both the co-activation and reciprocal activation are changed in order to tune the time varying patterns of muscle activation. Through the modification of the patterns of muscle activation, both the joint torque and joint stiffness are changed to produce the described mechanical behavior at the endpoint of the limb.

**Figure 1:**
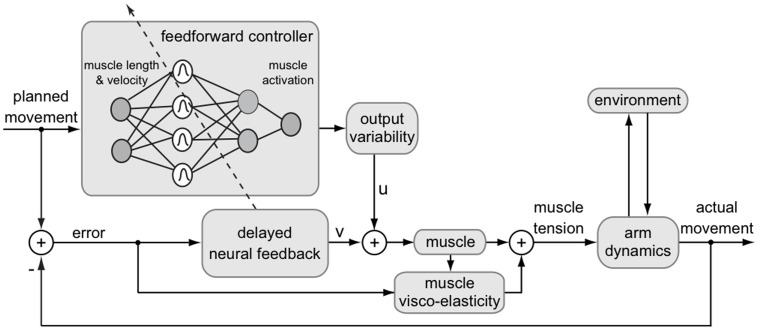
Diagram of human motor control model with a radial-basis function neural network learning feedforward muscle activation formulated in muscle coordinates.

Here we extend this previous work by formulating a model of human motor adaptation at the muscle level capable of trial-by-trial learning and generalization across multiple movements. In order to produce this generalization behavior, corresponding to experimental results, the feedforward motor command cannot simply be learned as a function of time for only a single movement as in our previous version [21], [22], but is learned as a function of the state of the limbs (joint position and velocity). In this manner, learning experienced in one movement will generalize both to neighboring movements and to any movement that experiences similar states of the limb. In order to adapt the algorithm to a state-space implementation, it was necessary to explicitly derive an algorithm from the principles behind the V-shape adaptation law. We derived this algorithm by translating the principles of motor adaptation from [21], [22] to arbitrary movements and motor primitives. Adaptation was formulated as the gradient descent of a cost function of error and effort, where the error in a muscle results both from its stretch or shortening, corresponding to experimental data [21], [23]. The adaptable feedforward control was then implemented as a radial basis function neural network (Fig. 1).

Our model does not attempt to reproduce the exact physiology from cortex to muscles, but reflects important functional properties that have been observed in sensorimotor learning. Notably, the model reproduces the fact that sensorimotor adaptation is generalized to neighboring movements [2], [6], [9]. This is produced by using a radial basis function network with local activation fields. Simulations systematically test whether the model reproduces experimental results, and revisit representative psychophysical studies of generalization in motor learning [2], [3], [24]. We simulate generalization to a variety of movements with distinct dynamics [2], and investigate learning for both fine grained force fields [3] and multiple movements with lateral instability [24].

## Methods: Novel Model of Human Motor Adaptation

### Motion control

Human joints are actuated by a redundant set of muscles, each of which can only pull. The activation of these muscles produces torques on the joint. Co-activation of antagonist muscles results in the canceling out of the joint torques weighted according to the moment arms. However, co-activation also contributes towards increasing joint impedance as the impedance of each muscle increases with activation [25], and impedance adds in all muscles spanning a joint [26].

How do humans use these muscle properties to adapt to novel environments? The observations of learning patterns in [20], [23], [27] suggest *principles of motor adaptation* which we have previously derived [21], [22]. The first principle states that the *motor command* for the 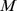 muscles involved in a movement, 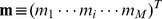, is composed of a *feedforward command* 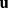 and a delayed *feedback command* 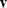 (Fig. 1):
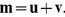
(1)Feedforward control is necessary to produce skillful movements in the presence of instability and interaction forces despite delays in the neural system. Feedback control, corresponding to the restoring force of the muscles once a disturbance is present (i.e., 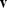 is defined by Equ.(1)), acts to stabilize motion. When the human hand is slightly perturbed during arm movements, it tends to return to the undisturbed trajectory [28] due to the instantaneous effects of muscle elasticity, short and long latency stretch reflexes, and involuntary visuomotor responses [29], [30]. However, 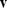 is not strictly equal to neural feedback, as descending feedforward commands can modulate neural reflexes and other feedback contributions to the overall motor response, and these feedforward/feedback interactions are likely to be highly nonlinear outside of a local operating range.

In the multi-joint limb system, long latency reflexes do not always inhibit the shortening muscles. Indeed, there are a variety of studies which show excitation in response to muscle shortening at delays within normal reflex latencies when stability is important [31]–[34]. Furthermore, feedback responses on initial trials in novel force fields also express this co-activation of antagonist muscles [21]. Corresponding to this experimental data, for each muscle 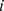, feedback 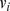 in Equ.(1) is an increasing function of both stretch and shortening, with a larger slope for stretch. For simplicity we assume that this function is linearly increasing in both directions, i.e.
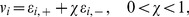
(2)where 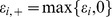 is the positive part and 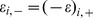 the negative part of the feedback error
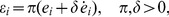
(3)where
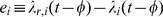
(4)is the difference of muscle length 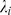 to the reference length 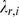 and 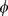 is the delay of neural feedback. Through equation (3), we assume that a filtered version of muscle length error (comprising the change in muscle length and muscle velocity relative to an undisturbed trajectory), is available to the CNS through afferent feedback such as muscle spindles [35]. These muscular error signals could arise either directly from the afferent feedback from the muscles if the gamma motor drive contained information about the predicted future state of the joint [36] or could arise in the central system through comparison of the afferent feedback with the predicted sensory consequences of the performed action. Through equations (2–4) the two sides of the V-shape function used for the learning law in [21], [22] are now contained within the feedback error signal used to drive the adaption. As a consequence learning will now correspond to an extended *feedback error learning* [12], able to deal with both adaptation of force and impedance in a unified way. Although it is not required that such error signals driving increased muscle activation in both agonist and antagonist muscles also appears in the feedback motor output (stretch reflex responses), there are many examples of such behavior in motor control [32]–[34].

### Minimization of error to keep stability with minimal effort

We assume that the feedforward motor command 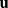 depends on positive activation parameters 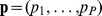. For example, if 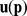 is represented by a neural network specifying how the feedforward motor command depends on the state of the limbs, these parameters would correspond to the weights of this network. 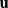 may be a linear function of the parameter vector 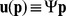, where the matrix 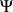 can be a linear function of the state (e.g. in a Perceptron neural network) or a nonlinear function (e.g. in a Gaussian radial basis function neural network). The other motor adaptation principles from [21] yield that motor learning consists of adapting the activation parameters in order to minimize movement error and effort, which we express as the cost function:
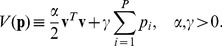
(5)

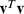
 is a cost for movement feedback error and 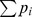 for the activation, i.e. for feedforward activity and consequently impedance.

We assume that learning corresponds to the gradient descent minimization of the cost function Equ.(5), i.e. activation is updated proportionally to the gradient of this function:
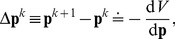
(6)where 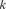 is a trial index. The gradient descent update of cost Equ.(5) is
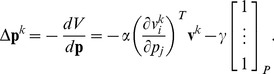
(7)In Equ.(1) 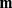 should ultimately represent the environment to learn and is thus assumed to be independent of 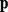, i.e. 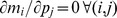. Equ.(7) then yields:
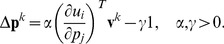
(8)


The second term 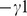 of Equ.(8), producing the same decrease of activation in all parameters 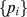, is minimizing the overall activation and thus impedance in a subtle way. If activation 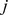 is smaller than activation 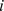, then 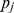 is decreasing relatively faster than 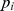. This enables learning law Equ.(8) to realize a winner-take-all scheme selecting the activation directions that were increased most from 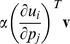. In the initial trials, the feedback error is large and most of the activation modification results from 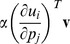. Later in the learning, optimization of impedance is performed from the term 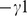, producing a decrease of impedance in the directions less activated, i.e. the direction of small impedance.

Let the motor command be of the form:
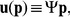
(9)where 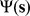 is a matrix of possibly nonlinear functions of the state 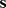 over some unknown *state space* spanning for example joint or muscle position and velocity. This linear function of activity parameters can be used to model many biological and artificial systems, including the rigid body dynamics model of serial or parallel mechanisms [37], nonlinear adaptive control [38], neural networks (as shown below), force fields [39], [40], time-varying and synchronous muscles synergies [41], [42] and linear superpositions of differential equations [43]. Gradient descent of cost function Equ.(5) yields then the *learning law*

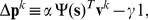
(10)where 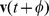 compensates for the feedback delay. Sensitivity analysis of the algorithm [22] has shown that that the model is robust to long delays up to 200 ms and exhibits how the learning parameters influence performance. Note that the update of each activation parameter depends only on the error, and is independent on the other activations, i.e. no explicit dependence between the activations is needed to regulate endpoint force and impedance with the coupled and highly nonlinear dynamics of the redundant neuro-muscular system.

We recognize in Equ.(10) the term 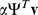 of feedback error learning [12] or traditional nonlinear adaptive control [44]. However, this relation is now in muscle space instead of non-redundant joint or hand space for adaptive control. This formulation means that stochastic deviation from the mean trajectory over consecutive trials results in increasing co-activation, and thus impedance, providing improved control [22]. In addition, while adaptive control is only concerned about identifying parameters that ensure the best tracking of the desired trajectory, the novel algorithm concurrently minimizes activation (Equ.(5)).

With the asymmetric V-shaped feedback error Equ.(2), this adaptation law can be decomposed as:
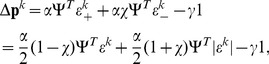
(11)where
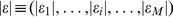
(12)is defined component-wise. It was shown in [21] that a deviation to one direction is compensated for by a force in the opposite direction in the next trial, hence 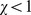. In this representation, the first term in 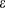 produces a force opposed to the error, i.e. compensates for systematic error, the second term in 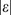 increases co-activation in response to deviation, i.e. increases stability, and the third term 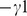 removes superfluous (co-)activation. Therefore, *the adaptation of Equ.(10) (or Equ.8) concurrently decreases instability, movement error and effort*.

The first term of Equ.(11) produces a modification of reciprocal activation, and corresponds to the force regulation algorithms of nonlinear adaptive control [44], iterative learning control [45], and previous models of motor learning [12], [13], [38]. The other terms tune the co-activation in all antagonist muscles groups, i.e. our scheme is extending these algorithms to simultaneous regulation of force and impedance. Recent robotic implementations of this controller [46], [47] have demonstrated its efficiency to producing an adaptive motor behavior with force and impedance tuned to the environment.

### RBF neural network feedforward model

As the neuromuscular system has to perform a range of movements in the dynamics of unknown environments, we model the feedforward command 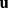 in Equ.(1) as a mapping which will be adapted during motion. We represent this mapping as a radial-basis function neural network described by (Fig. 1):
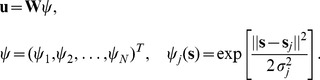
(13)where 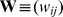 are the parameters or *weights* of the neural network which are adapted during learning. 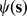 are Gaussian functions representing 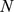 *neurons*, with the state space vector as input, whose components are both position and velocity: 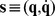. Each neuron 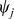 is characterized by its *centre* 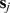 in the state space and its *activation field* 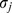.

To derive the learning law, we first need to set the neural network of Equ.(13) in the format of the linear model of Equ.(9):
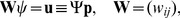
(14)thus
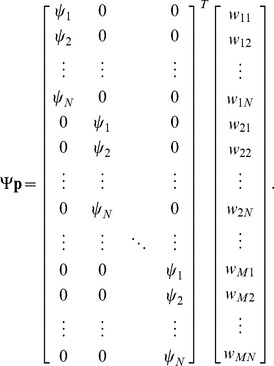
Using this identity, it can be shown that the learning law Equ.(10) yields
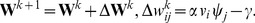
(15)


**Figure 2:**
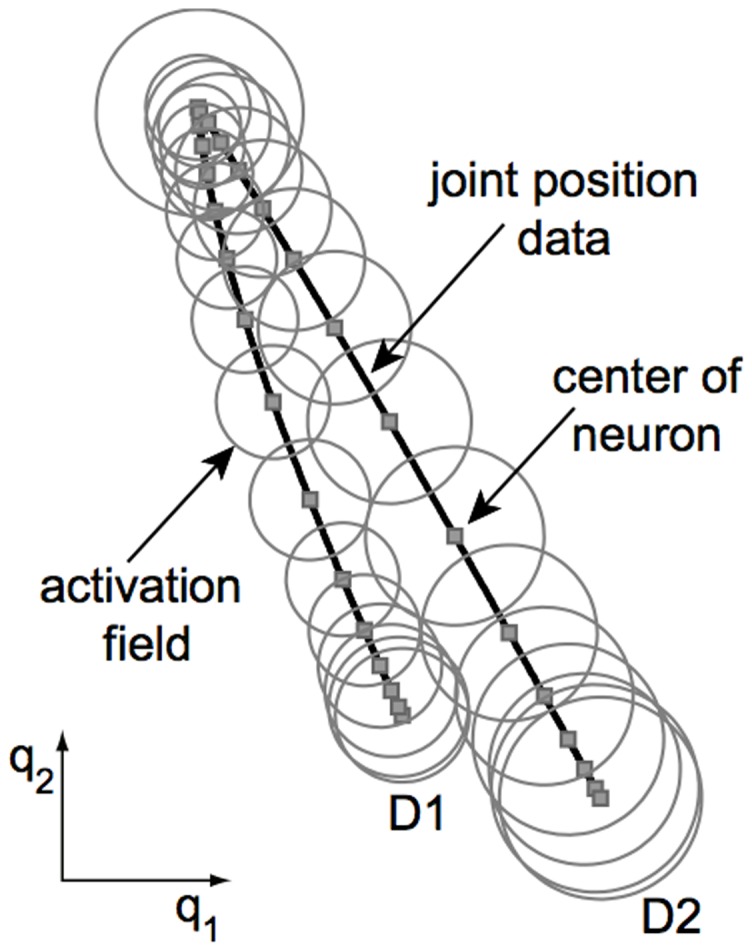
Illustration of data clustering for a two-dimensional joint space using *K*-means algorithm (as described in the “Novel model of human motor adaptation” section). The two paths correspond to the movements of Section “Impedance learning in multiple movements” (however the simulations of that section are done with a neural network over a four-dimensional state space 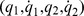).

To assign neurons to a group of data, we use a simple unsupervised learning algorithm called *K*-means [48], [49], which minimizes the sum of the square distances to the neurons centers:
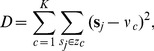
(16)where 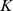 is the number of clusters, 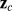 the vector of clusters where each cluster is assigned a centroid 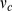 which is the mean point of neurons centers 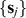 in this cluster (Fig. 2).

## Results: Simulation of Generalization in Force and Impedance Learning

The above model was used to simulate representative generalization experiments from the literature [2], [3], [24] and test hypotheses on motor adaptation formulated in these papers. The simulation used the two-joint six-muscle biomechanics structure of [22]. Multijoint arm movements with minimal jerk hand trajectory were used for the simulations of the next two paragraphs. For the simulation of movements with lateral instability requiring the variability of human movements, free movements from [24] were used. The difference from the mean trajectory was used as motion error for on-line control as detailed in [18]. Learning was performed in various force fields as described below. The computation steps are summarized in Table 1.

**Table 1:** Operations used at each time step to simulate the human arm learning a force field.

Function	Algorithm	Description
Computation of motor	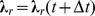	new reference length
command in the CNS	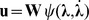	feedforward command
	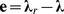	tracking error
	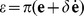	sliding error
	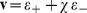	neural feedback
Arm musculature	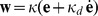	visco-elasticity
	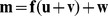	muscle tension
	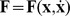	force field
	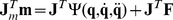	motion integration
Learning in the CNS	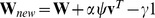	feedforward update

**Details and variables definitions can be found in the paper and in** [**22**] **.**

Muscle elasticity, neural feedback and signal dependent noise were modeled using the physiological parameters described in [22]. A feedback delay 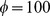 *ms* was assumed. To initialize the neural network by assigning centroids to the clusters in the task space, the *k*-means algorithm implemented in Matlab (the Mathworks Inc.) was used. For all experiments, the number of neurons was allowed to vary between 3 and 80, and the algorithm was initialized with 20 neurons. 2000 iterations were performed yielding 22–40 neurons in the experiments of this study.

The *K*-means algorithm assumes that the number of clusters 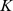 is known and there is an initial guess of the centre of each cluster. The conventional *K*-means clustering algorithm can in general only achieve local optimization to a solution that depends on the initial locations of cluster centers. Therefore, we let the algorithm run a number of times with the cluster centers at different locations, and selected the locations with minimal *D*. After calculating the number and centers of neurons, the activation fields of all neurons 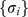 were chosen to include all data within the cluster, and are multiplied by a positive scaling factor such that the Gaussians of adjacent neurons overlap and ensure a smooth transition across data. Fig. 2 illustrates how the *K*-means algorithm clusters data from two straight line movements separated by 35°, using joint positions (as is used in Section “Impedance learning in multiple movements”).

### Movement generalization

**Figure 3:**
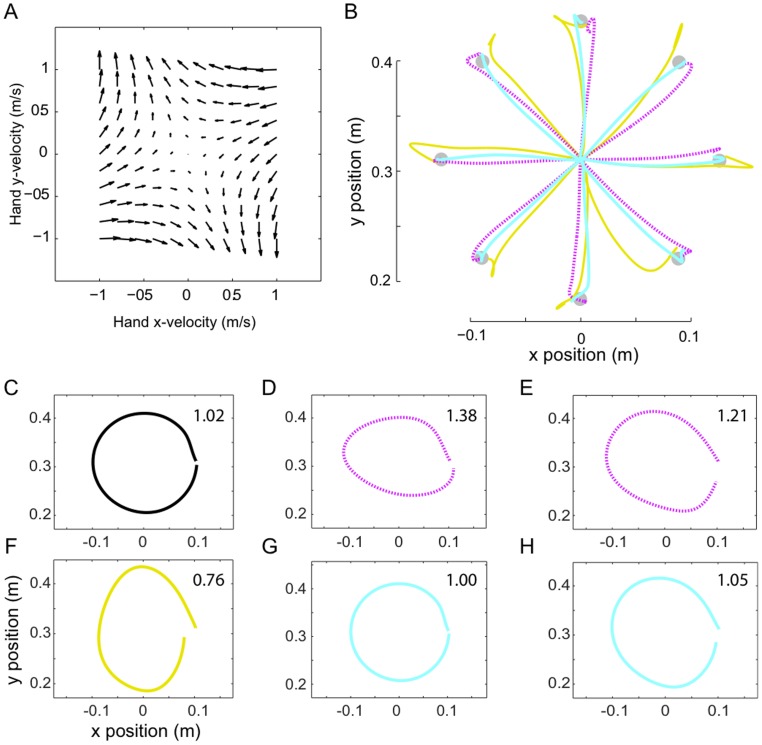
Simulation of transfer of learning to other movements as in [**2**] (compare with **Fig. 4** of [**2**]). Learning directly on a circle (C,D,F,G) or training reaching movements in all directions (B,E,H) results in similar performance and after effects. A: Velocity-dependent force field. B: Reaching movements in eight directions show early learning trials in the force field (yellow), late learning trials in the force field (cyan), and after effect trials when the force field was removed (magenta). C: Circle drawn in the null field before the simulation experiences the force field. D: After effects of adapting to the force field directly while making circles. E: Transferred after effects from learning the force field with reaching movements (as shown in B) to circular movements. F: Circular movements performed by the simulation during the initial unexpected exposure to the force field before learning. G: Circles drawn after adaptation to the VF directly by making circular movements. H: Transferred learning from reaching movements in 8 directions (as shown in B) to the circular motion. The number in the top right of each panel C-H indicates the ratio of horizontal to vertical axes of the drawn circle.

Generalization of dynamical learning to different movements was demonstrated by [2]. The experiment involved adapting to a velocity dependent force field VF (Fig. 3A) defined by:
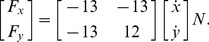
(17)One group of subjects learned the VF by performing reaching movements to randomly selected targets in 8 directions separated by 45°, after which the transfer of learning to a circular movement in the same force field was investigated. A second group directly learned to perform circular movements in the VF. Both groups of subjects exhibited similar abilities to deal with the dynamics despite different training regimes. Moreover, after effects from the adaptation were similar for both groups [2].

In order to simulate the experiment by [2], the RBF network was first initialized in the interaction free condition using joint position and velocity data from eight 12 *cm*-long movements spanning a range of 360° and a 10 *cm* radius circle. Both reaching movements and circular movements had duration of 300 *ms*. This neural network was then used to simulate the learning of movements.

Results of learning with 100 reaching movements in the eight directions are shown in Figure 3B. In *before effect trials*, i.e. initial exposure to the force field (yellow), movements diverge according to the force field. After adaptation to the external dynamics (cyan) movements become similar to those in NF. *After-effects trials* (magenta) slightly deviate opposite to the before effects trials, confirming the development of a learned compensation for the force field [1].

Figures 3C–H show similar results as in [2]. In the free condition, the simulation is able to make circular movements (Fig. 3C) which are disturbed when the force field is introduced (Fig. 3F). After training, the simulation is able to make undisturbed circular movements in the force field (Fig. 3G). Fig. 3D shows the effect of removing the VF after learning. The flattened circular after effect illustrates the learned feedforward control. Interestingly, similar results are obtained when the dynamics of the force field are trained with reaching movements rather than circular movements, corresponding to the first group of subjects in the human experiment. Fig. 3H shows the path after learning and Fig. 3E the after effects.

The circular deformation can be examined from the ratio between the horizontal and vertical axes (the numbers in the top corners in the panels of Fig. 3C–H). Movements in the VF after learning either along the circle (H) or the reaching movements (G), exhibit a ratio of approximately 1, as in the free condition (C). Initial trials in the VF (F) have ratio 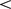
0.8 showing the vertical elongation, while all after-effects have ratio 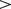
1.2 whether learned along the circle (D) or in reaching movements (E). This demonstrates a transfer of learning from the reaching movements to the circle, and illustrates that the feedforward control learned by performing reaching movements in VF is valid for different movements requiring distinct dynamics, i.e. *the generalization property of the model*.

### Granularity of the feedforward model

**Figure 4:**
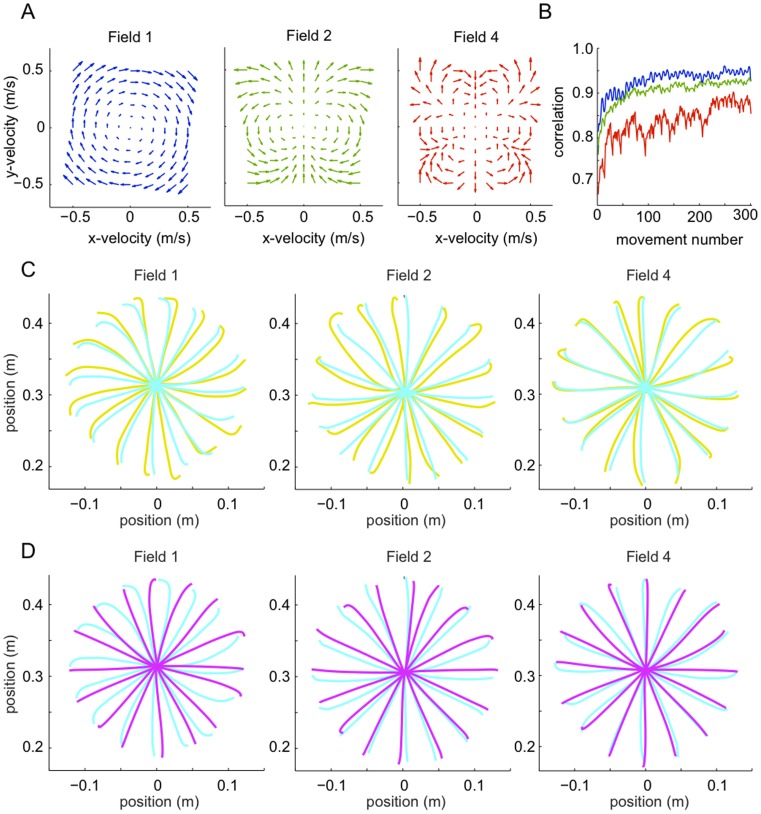
Simulation of reaching movements in force fields of increasing complexity as in [**3**]. A: Velocity dependent force fields VF1, VF2, VF4. B: Evolution of correlation between velocity profiles of movements in VF and the NF during learning. C: Movements performed before learning (in yellow) and after learning (blue) in the force fields. D: Movements after learning (blue) and during after effects trials (magenta) (where the force field is removed after learning).

Thoroughman and Taylor [3] investigated how much of the complexity of force fields can be learned. Subjects made reaching movements in 16 directions from 0° to 337.5° separated by 22.5° while the robotic arm disturbed the hand in increasingly variant velocity dependent force fields VF1, VF2, VF4, described by:
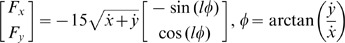
(18)where 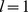 for VF1, 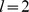 for VF2 and 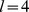 for VF4, the force is in *N* and the velocity in *m/s* (Figs. 4A). The experimental results showed that learning was possible for fields of various complexity, though the most complex field was not learned as well as the other two. A neural network model with adjustable size of activation fields was shown to account for these results, and it was thus concluded that the brain coding may adjust the size of the activation fields as the field complexity (how quickly the forces change in state space) increased.

In our simulation, 12 *cm* long movements of duration 300 *ms* were made to targets which were randomly presented in one of the 16 directions. 80 free movements were first performed followed by 160 movements in either of VF1, VF2 or VF4. The neural network was created with both position and velocity input data for all 16 directions of movements. The number of neurons and their corresponding activation fields were identical for adaptation to all three force fields. The force field defined in Equ.(18) was implemented with movements randomly simulated towards each of the 16 targets.

Results of our simulations show that the controller was able to learn the dynamics in all three force fields, but did not produce similar learning in VF4 as in the other two fields. Fig. 4D exhibits large deviation of the after-effects in VF1 and VF2 relatively to learned movements, providing evidence for a modification of the feedforward control, however there was little deviation in VF4. Furthermore, the learned feedforward command can efficiently reduce the effect of the respective dynamics in VF1 and VF2 (Fig. 4C), while in VF4 the trajectories after learning do not deviate much from the before-effects. Quantitative analysis of the learning is provided by the correlation of the velocity time series between free movements in all directions and respective movements after learning in VF1, VF2 and VF4. The correlation was computed as in [1] and smoothed using a 20-movement moving average. We see in Fig. 4B that the movements in VF1 and VF2 become increasingly well correlated with the free movements. In VF4 the correlation evolution is more volatile. It also increases but remains below 0.9.

In summary, the simulation yields similar results as in the experiment of [3]. That is, our model reproduced all of the major features of the experimental results, without varying the neural coding from one environment to another. Specifically, all three force fields were learned by the simulation, however the most complex environment (VF4) was not learned as well as the other two, exactly as found by the experimental results. This suggests that there are clear limits in the ability of this internal model in learning fine granularity of changes in the external environment. Thus a single neural coding was able to produce a feedforward activation model able to deal with the fine granularity of all three external environments. In contrast to the interpretation of [3], it was not necessary to modify the size of the neural activation fields which code the feedforward activation model in order to match the force field spatial complexity. However, agreeing with the experimental results [3], the approximation property of the internal model was limited, such that VF4, with its fine granularity, could not be learned well. Together the simulation and experimental results indicate a clear tradeoff or interference between the generalization of the learned model (how well learning at one point can be used to nearby state spaces) and the specificity of the internal model (how fine grained the learned force compensation could be).

### Impedance learning in multiple movements

While many studies have examined the generalization functions of adaptation to stable environments, e.g. [1]–[3], [8], almost all studies examining adaptation to unstable dynamics have focused on a single movement direction [23], [50]–[52]. Recently, we examined adaptation to two separate movements, separated by 35°, to unstable environments oriented perpendicular to the direction of each movement [24]. The results showed that subjects were able to learn to selectively increase endpoint stiffness in each movement only in the direction of instability and switch between these on a movement by movement basis. Therefore in order to test the capability of our model for impedance learning and unstable tasks we simulated the task of [24]. In that experiment, subjects performed reaching movements with lateral instability to any of two targets separated by 35°. Lateral instability was produced by a position-dependent divergent force field (DF) of the form:
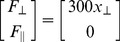
(19)where 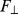 and 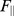 indicate the components of the force applied on the hand (in *N*) normal and parallel to the straight line from start to end points, respectively and 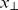 is the lateral deviation of the hand from this straight line. A virtual safety barrier was implemented when the hand deviated by more than 5 *cm* from the straight line between start and finish points, consisting of large damping replacing the negative stiffness of Equ.(19). There was no force field inside the (2.5 *cm* diameter) start and end circles.

**Figure 5:**
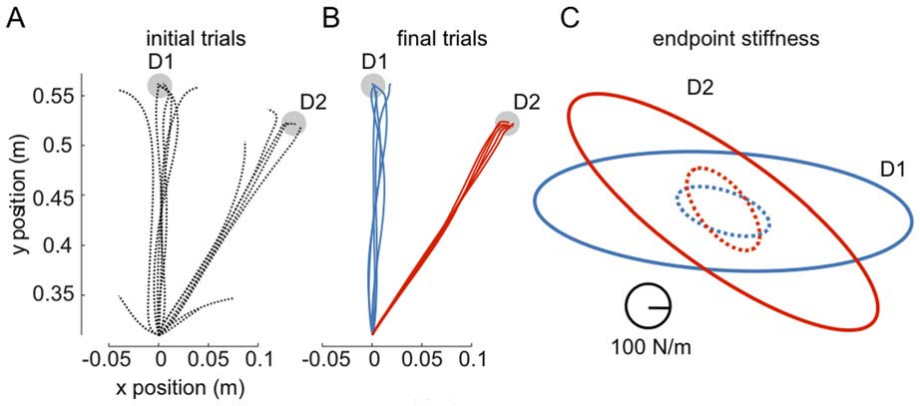
Learning movements to multiple directions in unstable dynamics. Initial trials in the divergent field for both D1 and D2 (A) and movements after learning 300 trials (B, D1 in blue and D2 in red). C: Stiffness ellipses 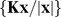 (where 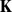 is the 2×2 endpoint stiffness matrix [22]) before (dashed line) and after (solid line) learning in D1 (blue) and D2 (red).

We simulated 600 *ms* long point-to-point movements from 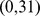 *cm* relative to the shoulder to either 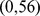 *cm* (called D1), or 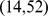 *cm* (D2), which were randomly intermixed (Fig. 5). The simulation results of hand trajectories and endpoint stiffness during adaptation to DF are similar to experimental data [24]. The model was able to adapt to the instability in both D1 and D2 directions simultaneously. Initial trajectories deviated to either the right or the left of the straight-line trajectory (Fig. 5A), but with adaptation the model produced nearly straight movements to the target similar to free movements (Fig. 5B). Stiffness was computed from the muscle activation as described in [18] which incorporates contributions to endpoint stiffness from both the feedforward and feedback components of muscle activation.

Figure 5C shows the endpoint stiffness ellipse after learning in DF relative to the free condition. After learning in DF stiffness increased in size for both D1 and D2 movements, and the stiffness ellipse elongated in the respective direction of instability as was found in experiments [24], [50]. Note that while in NF the stiffness was already elongated lateral to the movement direction, however in DF the increase is much larger in the lateral movement direction as along the movement direction, compensating for the force field instability.

These results show that our model is capable of learning to perform multiple movements with lateral instability, as is required in many tasks with tools, e.g. carving. Similar to the human experimental results, the simulation learned a behavior needing minimal force and impedance, resulting in a stiffness ellipse elongated in the direction of instability and compensating for it. Force and impedance required for each movement could be learned easily, without interference with other movements, as was found experimentally [24], [53], [54].

## Discussion

This paper introduced a model of human motor adaptation able to learn multiple movements in stable or unstable dynamics, and to generalize this learning across state space, extending the learning formulation of [21], [22] to multiple movements and generalization. In this novel version of the algorithm, adaptation arose from the gradient descent minimization of a cost function corresponding to feedback and feedforward muscle activation. A mapping from state space to muscle activation coded in a radial basis function network was gradually identified during movements, yielding a model of feedforward control valid in this state space.

Previous models based on iterative control or adaptive control algorithms [7], [8], [12]–[16] predicted the evolution of force, but did not consider motor output variability and could not adapt to instability as they did not have a mechanism to adapt impedance. Furthermore, these models - when perturbed by a velocity dependent force field - predict only an increase in the activity of the stretched muscle, with no increases in co-contraction such as found experimentally [20], [27], [55], [56]. On the other hand, our model predicts the appropriate changes in both the stretched and shortened muscles resulting in increases in co-activation due to any perturbing inputs to the limb. Models based on optimization [10], [11], [57]–[62] compute the coordination of processes, muscles and limbs in one step, yielding only the post-learning optimal behavior. In contrast, our algorithm is able to predict the trial-by-trial changes of muscle activation, which may predict distinct behaviors resulting from convergence to local minima or to bifurcations [63], and is critical for use in neurotechnology applications such as neural prostheses.

A major conceptual difference from our previous work [21], [22] is the incorporation of the V-shaped function, which gives rise to simultaneous changes in impedance and force, into the neural feedback command. The new model proposed here uses neural feedback as a V-shaped function of kinematic error, providing increased feedback co-activation in response to perturbations. This modified neural feedback corresponds to the results of several studies that have illustrated that when stability requirements are important, perturbations of the limb give rise to increases in the muscle activity of both the stretched and shortened muscles [31]–[34]. Incorporating the V-shaped function into the feedback command enabled us to form a cost function of the feedback and effort. Gradient descent on this cost function gives rise to an adaptation law that can be used for a variety of motor primitives, such as muscle synergies, physical models, neural networks and linear families of differential equations. The new algorithm extends feedback error learning [12] in that both force and impedance are adapted simultaneously, using an adaptation law that corresponds to observed experimental changes in muscle activation.

The simulations of multijoint movements in this paper, together with the results from [21], [22], demonstrate the efficiency of this nonlinear adaptive controller. The algorithm derived and implemented in this paper learned to perform movements in any direction in either stable or unstable dynamics despite motor output variability. Moreover, it was able to generalize from one movement to another, and converged to suitable force and minimal impedance behavior. This novel computational model learns to coordinate motor commands without requiring any inversion or any model of the actuators. It can be applied in iterative control along a single movement [22] or on a periodic trajectory, to identify the parameters of a known dynamic structure in the sense of adaptive control [44], [64], or to learn muscle synergies or unstructured dynamics with a neural network (as was shown in this paper).

These characteristics of the ‘human controller’ are very attractive for robotic systems interacting either with the environment or with humans. To our knowledge, there exists no previous robotics algorithm that is able to control force and impedance and acquire stability. Some of the possibilities of this learning controller have been demonstrated in recent implementations with a seven degrees of freedom industrial robot and with a novel variable impedance actuator [46], [47]. This controller can be easily implemented on a robot providing an automatic adaptive motor behavior that learns stable performance in interaction with the environment with the appropriate force and impedance [46], [47]. An interesting feature of human learning is that impedance is increased with any movement error [65]. Therefore, in presence of any perturbation, the controller first increases impedance, making the control robust, before adapting the force in order to perform the task successfully while relaxing impedance.

From the neurophysiological point of view, the simulation suggests that to compensate for external dynamics, the sensorimotor control system needs to consider the limb state in order to compute the appropriate feedforward motor command. The computational model was able to predict the generalization patterns observed during learning of multiple movements by humans [1], [3], [24], and across movements requiring different dynamics [2]. It predicted correct patterns of force, impedance and muscle activations in multiple directions [24] and in various dynamic environments [22]. Compared to experimental work [3], the model also found similar limitations in the granularity of dynamic environments that it was able to compensate for. However, in contrast to the analysis of [3], our results suggests that neurons are not required to adapt their activation fields in order to reproduce the experimental results.

As currently specified, the algorithm adapts to the environment through learning of the appropriate feedforward motor command. However, sensorimotor learning does not occur purely through adaptation of the feedforward motor command, but also through the modulation of the feedback responses, tuning them to the environment (see [66], [67] for reviews). That is, the gain of the feedback responses have been shown to modulate early in learning [55], after adaptation to stable [55], [68] and unstable dynamics [52], [69], and depending on the relevance of the perturbations to the overall task [30], [34], [70]. Such changes in feedback control are not explained within our current algorithm which focuses purely on the learning of the feedforward motor command.

In summary, the model described in this paper is an elegant solution to motor adaptation, relying on biologically plausible signals and resulting in skillful motor behavior. The novel algorithm, without an explicit model of either the impedance or the force, learns in a single process the time-varying motor commands that result in the appropriate force and tuned mechanical impedance. The properties of motor generalization and dynamic coupling between the muscles emerge through the learning process.
